# From allergy to arrhythmia – electrophysiological basis for the antiarrhythmic properties of antazoline

**DOI:** 10.3389/fphar.2026.1780526

**Published:** 2026-03-27

**Authors:** Felix Wiedmann, Anna Prodanova, Manuel Kraft, Frederike Marie Weitkamp, Amelie Paasche, Christian Goetz, Constanze Schmidt

**Affiliations:** 1 Department of Cardiology and Pneumology, University Medical Center Goettingen, Goettingen, Germany; 2 DZHK (German Center for Cardiovascular Research), Partner Site Lower Saxony, University of Goettingen, Goettingen, Germany; 3 Department of Cardiology, Angiology and Pneumology, Heidelberg University Hospital, Heidelberg, Germany; 4 DZHK (German Center for Cardiovascular Research), Partner Site Heidelberg/Mannheim, University of Heidelberg, Heidelberg, Germany; 5 Institute of Biomedical Engineering, Karlsruhe Institute of Technology (KIT), Karlsruhe, Germany

**Keywords:** action potential, antazoline, antiarrhythmic drugs, atrial fibrillation, multichannel inhibitor

## Abstract

**Introduction:**

Antazoline, a first-generation H_1_-antihistamine, has shown rapid and effective antiarrhythmic action, particularly in recent-onset paroxysmal atrial fibrillation. Despite clinical use in some countries, the underlying electrophysiological mechanisms remain incompletely understood, and concerns about potential proarrhythmic effects persist. Here, we aim to systematically characterize the ion channel interaction profile of antazoline and assess its molecular mode of action.

**Methods:**

Electrophysiological measurements were performed using the two-electrode voltage clamp technique on 22 cardiac ion channels, expressed in *Xenopus laevis* oocytes.

**Results:**

Antazoline strongly inhibited hERG (IC_50_ end-pulse: 43.3 µM; peak-tail: 162.6 µM) and hKir3.1/3.4 (IC_50_: 52.8 µM) in a concentration- and state-dependent manner. Block of hERG was attenuated by Y652A and F656A pore mutations, implicating classical aromatic binding residues. Positive rate dependence and open- and inactivated-state as well as partial closed-state inhibition were observed. In contrast, hK_2P_17.1 was significantly activated.

**Discussion:**

Antazoline exhibits a distinct multichannel profile in a heterologous expression system, combining potent inhibition of hERG and hK_ir_3.1/3.4 with activation of hK_2P_17.1. The combined modulation of these channels suggests a potential atrial-preferential electrophysiological profile, while hERG inhibition indicates a need for careful evaluation of ventricular repolarization safety.

## 1 Introduction

Atrial fibrillation (AF) is the most common sustained arrhythmia encountered in clinical practice, affecting nearly 60 million individuals worldwide (Li et al., 2022). Driven by increasing life expectancy and the growing prevalence of associated risk factors such as hypertension, obesity, and diabetes (Dong et al., 2023) the burden of AF continues to rise. Severe complications, including stroke, and heart failure, and cognitive decline substantially impair quality of life and contribute to elevated morbidity and mortality (Wolf et al., 1991; Wang et al., 2003). Consequently, AF imposes a major challenge not only on individual patients and treating physicians but also on healthcare systems globally.

Current guideline-based management of AF centers on rate or rhythm control, anticoagulation, and the treatment of underlying comorbidities (Van Gelder et al., 2024). In recent years, randomized trials have demonstrated the superiority of early rhythm control over rate control in reducing cardiovascular events and delaying AF progression (Kirchhof et al., 2020; Hohnloser et al., 2009; Camm et al., 2011). Pulmonary vein isolation (PVI) has emerged as the most effective rhythm control strategy. However, its real-world applicability is limited by infrastructure, cost, and procedural risks as well as high AF recurrence rates of 40%–50% within 5 years post PVI (Andrade et al., 2021; Kuck et al., 2021; Mark et al., 2019; Morillo et al., 2014). Pharmacological rhythm control, on the other hand, remains hampered by modest efficacy, proarrhythmogenic potential, and limited safety in patients with structural heart disease (Kovacs et al., 2023; Naksuk et al., 2019; Vinson et al., 2018; Hohnloser et al., 1995; Chevalier et al., 2003). Although understanding of AF pathophysiology has significantly advanced and atrial-selective multi-target drug strategies have been proposed (Saljic et al., 2022; Podd et al., 2016; Wang et al., 2017) clinical translation remains challenging and the antiarrhythmic drug (AAD) armamentarium in daily practice has not substantially changed over decades, leaving current pharmacological strategies insufficient. This stands in contrast to the high clinical need for safe and effective antiarrhythmic drugs which, in times of growing emphasis on rhythm control, is greater than ever. Yet, the development of novel agents is hindered by high regulatory and financial barriers, discouraging pharmaceutical investment. Here, drug repurposing (re-evaluating approved substances for new therapeutic indications) offers an attractive, cost-effective alternative (Wiedmann et al., 2022; Hegyi et al., 2022).

Antazoline, a first-generation antihistamine formerly used for allergic conjunctivitis, has long been noted for its antiarrhythmic effects, which were first described as early as the 1950s (Abelson et al., 1980; McKechnie, 1952). Over time, accumulating clinical data have demonstrated its efficacy in the cardioversion of various supraventricular and ventricular arrhythmias, particularly paroxysmal AF (pAF) (Palimonka et al., 2020; Calvert et al., 2022). The AnPAF and AnProAF randomized controlled trials recently reported conversion rates of 72.2% and 63%, respectively, with antazoline injected intravenously, highlighting its rapid onset, high efficacy, and favorable safety profile (Maciag et al., 2017; Karwowski et al., 2024).

Despite these promising clinical results, the electrophysiological mechanisms underlying antazoline’s antiarrhythmic action remain poorly understood. Human studies have noted prolongation of the P wave, QRS complex, and QT interval, as well as an increase in atrial effective refractory period (ERP) following antazoline administration (Piotrowski et al., 2017; Bińkowski et al., 2018; Farkowski et al., 2019). Experimental models further suggest prolongation of both atrial and ventricular ERP, action potential duration (APD), and post-repolarization refractoriness (Ellermann et al., 2018; Frommeyer et al., 2017). These changes imply a modulation of cardiac ion channels, but a detailed understanding of the molecular targets involved is lacking.

To address this knowledge gap, the present study characterizes the ion channel profile of antazoline with a particular focus on cardiac potassium channels. By elucidating its electrophysiological actions and biophysical mode of action, we aim to clarify the mechanistic basis of antazoline’s antiarrhythmic efficacy.

## 2 Materials and methods

### 2.1 Molecular biology and RNA preparation

Complementary (c)DNA clones encoding human (h)K_2P_1.1 (GenBank accession number NM_002245), hK_2P_2.1 (EF165334), hK_2P_3.1 (NM_002246), hK_2P_9.1 (NM_016601), hNa_v_1.5 (NM_000335.5), rat (r)K_V_4.3 (NM_031739), hKVLQT1 (NM_000218.3) and hMinK/*hKCNE1* (NM_000219.6) were kindly donated by Steve Goldstein (Irvine, CA, USA). C. Spencer Yost (San Francisco, CA, USA) contributed hK_2P_18.1 cDNA (NM_181840). cDNAs of hK_2P_4.1 (EU978935), hK_2P_5.1 (EU978936), hK_2P_6.1 (EU978937), hK_2P_10.1 (EU978939), hK_2P_13.1 (EU978942), hK_2P_16.1 (EU978943), hK_2P_17.1 (EU978944), and hK_V_11.1 (human ether-a-go-go-related gene (hERG)) wild type (wt) cDNA (NM_000238) as well as hERG-Y652A and hERG-F656A point mutations were obtained from Dierk Thomas (Heidelberg, Germany). hK_ir_2.1 (U12507) and hK_ir_2.3 (NM_004981) was provided by Carol A. Vandenberg (Santa Barbara, CA, USA). Niels Decher (Marburg, Germany) supplied hK_ir_3.1 (NM_002239), hK_ir_3.4 (NM_000890.3), rK_V_1.4 (NM_012971), and hK_V_2.1 (L02840). hK_V_1.5 (NM_002234) was a gift from Barbara A. Wible (Cleveland, OH, USA). cDNAs were subcloned into expression vectors containing either a T7 or SP6 promoter to allow *in vitro* transcription. Plasmid DNA was amplified in *E. coli* DH5α (Invitrogen, Thermo Fisher Scientific; Waltham, MA, USA) and purified using the QIAprep Spin Miniprep Kit (Qiagen; Hilden, Germany). Linearization was performed using restriction enzymes (New England Biolabs; Ipswich, MA, USA) appropriate for each construct. Linearized DNA was purified with the Qiagen PCR Purification Kit. *In vitro* transcription was conducted using the mMessage mMachine T7 or SP6 Kit (Ambion, Thermo Fisher Scientific) following the manufacturer’s protocol. After lithium chloride precipitation, transcript integrity was confirmed by agarose gel electrophoresis, and RNA concentrations were determined spectrophotometrically (NanoDrop One Microvolume UV-Vis Spectrophotometer, Thermo Fisher Scientific). cRNA was diluted in nuclease-free water to the desired concentration for *Xenopus laevis* oocyte injection.

### 2.2 Oocyte preparation

Ovarian tissue was surgically harvested from adult female *X. laevis* frogs (*Xenopus*1; Dexter, MI, USA) under aseptic conditions following anesthesia with 0.15% tricaine solution (pH 7.5). Aseptic extirpation of the ovarian lobes was alternated between the left and right lower abdomen, with a maximum of four interventions per individual. After the final procedure, anesthetized frogs were euthanized via decerebration. Retrieved oocytes were manually dissected and enzymatically defolliculated by incubation in collagenase derived from *Clostridium histolyticum* (Collagenase NB 4 Standard Grade, 0.17 U/mg; Nordmark Pharma; Uetersen, Germany). Stage V–VI oocytes were selected under a stereomicroscope and microinjected with 0.276–104.74 ng/oocyte cRNA, depending on the specific channel. Injected oocytes were incubated for 24–72 h in oocyte storage solution containing 100 mM NaCl, 2 mM KCl, 1 mM MgCl_2_, 1.8 mM CaCl_2_, 5 mM 4–(2–hydroxyethyl)–1–piperazineethanesulfonic acid (HEPES), 2.5 mM pyruvic acid, and 50 mg/L gentamicin sulphate, adjusted to pH 7.7 with NaOH prior to electrophysiological recordings.

### 2.3 Electrophysiology

Whole-cell currents from injected *X. laevis* oocytes were recorded using the two-electrode voltage clamp (TEVC) technique. Borosilicate glass microelectrodes (GB100F-10; Science Products, Hofheim, Germany) were pulled with a P-1000 horizontal micropipette puller (Sutter Instruments; Novato, CA, USA). Electrodes were filled with a 3 mM K^+^ internal solution composed of 102 mM NaCl, 3 mM KCl, 2 mM MgCl_2_, 1.5 mM CaCl_2_, and 10 mM HEPES, adjusted to pH 7.4 with NaOH. The resulting tip resistance ranged from 1 to 3 MΩ. Macroscopic membrane currents were acquired using an OC-725C Oocyte Clamp amplifier (Warner Instruments; Hamden, CT, USA), digitized with a Digidata 1550A (Axon Instruments; Foster City, CA, USA), and analyzed using pClamp 10 software (Molecular Devices; San Jose, CA, USA). Signals were sampled at 2 kHz and low-pass filtered at 0.02–0.2 kHz. Leak currents were not subtracted. All recordings were performed at room temperature (20 °C–22 °C) under constant gravity-driven perfusion. The standard extracellular bath solution contained 101 mM NaCl, 4 mM KCl, 2 mM MgCl_2_, 1.5 mM CaCl_2_, and 10 mM HEPES, adjusted to pH 7.4 with NaOH. For alkaline pH-activated channels hK_2P_16.1 and hK_2P_17.1, the extracellular pH was adjusted to 8.5. For enhanced current amplitudes in hK_2P_1.1 and hK_2P_6.1 channels, a modified rubidium-containing extracellular solution was used (101 mM RbCl, 4 mM KCl, 2 mM MgCl_2_, 1.5 mM CaCl_2_, and 10 mM HEPES, pH 7.4), based on the stabilizing effect of Rb^+^ ions on the conductive state of the selectivity filter (Nematian-Ardestani et al., 2020).

**FIGURE 1:**
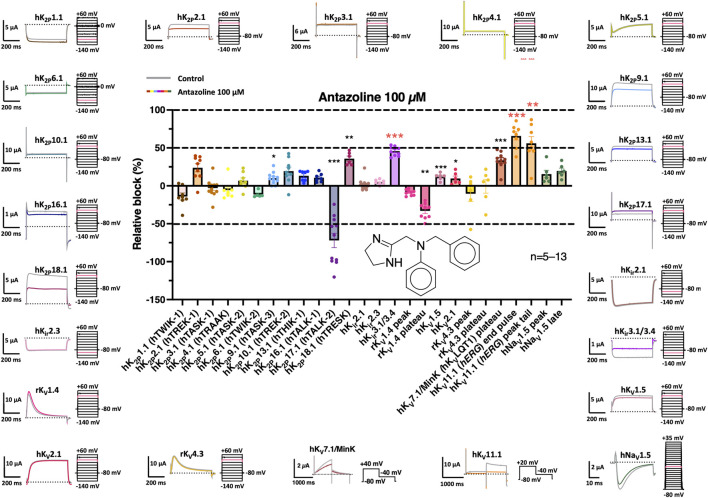
*Effects of antazoline on key cardiac ion channels heterologously expressed in Xenopus laevis oocytes.* Representative current traces are shown for the indicated channels under control conditions (grey) and following 30 min perfusion with 100 µM antazoline (colored), along with the corresponding pulse protocols. Test pulses associated with the presented trace are highlighted in pink. Dotted lines represent the zero current level and scalebars are provided as insets. *Center:* Relative current inhibition was quantified for each channel. Antazoline induced significant inhibition of hK_2P_9.1, hK_2P_18.1, hK_ir_3.1/3.4, hK_V_1.5, hK_V_LQT1/MinK and hERG channels, whereas hK_2P_17.1 and rK_V_1.4 exhibited significant activation. The chemical structure of antazoline is shown beneath the bar graph. Data are presented as mean ± SEM of *n* = 5–13 measurements; *, *p <* 0.05; **, *p <* 0.01; ***; *p <* 0.001 derived from two-tailed Student’s t-tests.

Excluding hK_V_7.1 (hK_V_LQT1)/MinK and hK_V_11.1 (hERG), all tested K_2P_, K_ir_, and K_V_ channels were stimulated using a standard voltage-clamp protocol consisting of 500 m depolarizing pulses from −140 mV to +60 mV in 20 mV increments at 0.2 Hz. For quantitative analysis, plateau current amplitudes at a test pulse potential of +20 mV were measured for hK_2P_2.1, hK_2P_3.1, hK_2P_4.1, hK_2P_5.1, hK_2P_9.1, hK_2P_10.1, hK_2P_13.1, hK_2P_16.1, hK_2P_17.1, hK_2P_18.1, rK_V_1.4, hK_V_1.5, hK_V_2.1, and rK_V_4.3. Peak currents at +20 mV were additionally evaluated for rK_V_1.4 and rK_V_4.3. For potassium channels with (partially) inwardly rectifying characteristics (hK_2P_1.1, hK_2P_6.1, hK_ir_2.1, hK_ir_2.3, and hK_ir_3.1/3.4), plateau current amplitudes were measured at −100 mV. For generation of the data depicted in Figure 1, hK_V_LQT1/MinK and hERG currents were elicited using a double-step protocol: from a holding potential of −80 mV, cells were depolarized to +40 mV (hK_V_LQT1/MinK) or +20 mV (hERG) for one second, followed by repolarization to −40 mV for another second at 0.1 Hz. For quantification, end-pulse and peak-tail current amplitudes were analyzed. For hNa_V_1.5, 40 m depolarizing pulses from −80 mV to +35 mV in 5 mV increments (0.2 Hz) were used, and both peak and late sodium currents were quantified at −25 mV. The holding potential was set to −80 mV for all channels, except for hK_2P_1.1 and hK_2P_6.1, which were held at 0 mV to reduce baseline leak currents. Unless stated otherwise, voltage pulse protocols were repeated every 2 minutes throughout the duration of the experiments. For all other experiments utilizing the heterologous expression of hERG K^+^ channels, protocols are to be found in corresponding figures and text legends.

### 2.4 Pharmacological compounds

Antazoline hydrochloride (Sigma-Aldrich; St. Louis, MO, USA) was dissolved in dimethyl sulfoxide (DMSO) as a 100 mM stock solution and stored at −20 °C. Aliquots of the stock solution were diluted to the desired concentration with the respective bath solution.

### 2.5 Statistical analysis and data visualization

Data acquisition and analysis were performed using pCLAMP 10 software (Axon Instruments; Foster City, CA, USA), Prism 10 (GraphPad Software; La Jolla, CA, USA), and Excel (Microsoft Corporation; Redmond, WA, USA). Concentration–response relationships for drug-induced block were fit with a Hill equation of the following form: *I*_*drug*_/*I*_*control*_ = 1/[1+(*D*/*IC*_*50*_)^*n*^], where *I* denotes the current, *D* is the drug concentration, *n* indicates the Hill coefficient, and *IC*_*50*_ is the half-maximum inhibitory concentration. Normalised I-V relations for hERG *I*_tails_ derived from peak-tail current amplitudes recorded during the repolarizing step to −40 mV were fitted with the following Boltzmann sigmoidal equation: *I* = *I*_max_ /[1 + e^(*V*50-*V*)/*k*^], where *V* is the test pulse potential, *V*_*50*_ is the half-maximal activation potential and *k* is the slope of the activation curve. For Boltzmann fitting, peak-tail currents were normalized to the maximal current amplitude obtained in each individual cell under the respective condition. Activation curves were then fitted on a per-cell basis using the Boltzmann equation, and resulting V_50_ values were averaged for statistical comparison. Data are expressed as mean ± standard error of the mean (SEM). Paired t-tests and multiple paired t-tests (two-tailed tests) with Dunn’s post-hoc test and Bonferroni correction for multiple testing were used to compare the statistical significance of the results where appropriate and *p <* 0.05 was considered statistically significant. Ordinary one-way ANOVA was performed for multiple comparisons. If the difference among group means reached *p <* 0.05 significance, pairwise comparisons were made, adjusted by the Tukey’s multiple comparisons test.

## 3 Results

### 3.1 Antazoline targets cardiac potassium channels

To evaluate the electrophysiological effects of antazoline on cardiac ion channels, sensitivity to antazoline was assessed across the full panel of functional K_2P_ channels, selected representatives of the K_ir_ and K_V_ channel families, and the cardiac sodium channel Na_V_1.5, following heterologous expression in *X. laevis* oocytes. After a stabilization period of ≥20 min in standard extracellular bath solution, oocytes were perfused with an antazoline-containing (100 µM) bath solution for 30 min, followed by a washout period of ≥20 min in pure bath solution.

The effects of 30 min perfusion with 100 µM antazoline on current characteristics are summarized in Figure 1. Significant current inhibition was observed for hK_2P_9.1 (human TWIK-related acid-sensitive K^+^ channel (hTASK) 3; 10.0% ± 2.4%; *n* = 11; *p* = 0.0435), hK_2P_18.1 (human TWIK-related spinal cord K^+^ channel (hTRESK); 35.8% ± 3.4%; *n* = 6; *p* = 0.0032), hK_ir_3.1/3.4 (45.8% ± 1.9%; *n =* 10; *p <* 0.0001), hK_V_1.5 (12.1% ± 1.8%; *n =* 6; *p =* 0.0307), hK_V_7.1 (hK_V_LQT1)/MinK (33.6% ± 3.5%; *n* = 10; *p* < 0.0001) and hERG (end pulse: 65.8% ± 5.5%, peak tail: 56.1% ± 8.6%; *n =* 8; *p*_end pulse_ = 0.0002, *p*_peak tail_ = 0.0085). In contrast, antazoline significantly activated hK_2P_17.1 (human TWIK-related alkaline pH-activated K^+^ channel (hTALK) 2; 72.0% ± 9.5%; *n* = 11; *p =* 0.0005) and rK_V_1.4 (peak: 8.9% ± 1.2%, plateau: 33.1% ± 3.4%; *n =* 9; *p*_peak_ = 0.0022, *p*_plateau_ = 0.0003). Given the pronounced inhibitory effects and their physiological relevance in cardiac repolarization, hK_ir_3.1/3.4 and hERG channels were selected for further in-depth pharmacological analysis with antazoline.

### 3.2 Concentration dependence of antazoline inhibition

**FIGURE 2:**
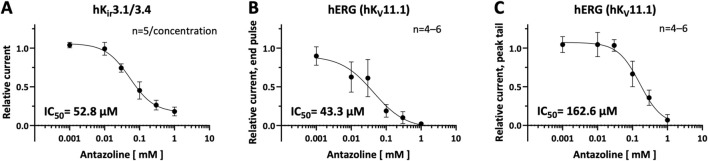
*Concentration–response relationships for antazoline-induced inhibition of cardiac ion channel currents.* Concentration–response curves were constructed by measuring the relative inhibition of current amplitudes following 30 min incubation with increasing concentrations of antazoline. Data were fitted using the Hill equation to determine the half-maximal inhibitory concentration (IC_50_) and Hill coefficient (*n*_*H*_). **(A)** hK_ir_3.1/3.4 currents: IC_50_ = 52.8 µM; *n*_*H*_ = −1.3 (*n =* 5 cells per concentration). **(B)** hERG end-pulse currents: IC_50_ = 43.3 µM; *n*_*H*_ = −0.9 (*n =* four to six cells per concentration). **(C)** hERG peak-tail currents: IC_50_ = 162.6 µM; *n*_*H*_ = −1.4 (*n =* four to six cells per concentration). Values are presented as mean ± SEM.

To characterize the concentration dependence of antazoline’s inhibitory effects on potassium channels, concentration–response relationships were determined for hK_ir_3.1/3.4 currents as well as for the end-pulse and peak-tail components of hERG currents (Figure 2). Current inhibition was measured at increasing antazoline concentrations of 1, 10, 30, 100, 300, and 1,000 µM after a drug-superfusion of 30 min. The corresponding voltage-clamp protocols used for these recordings are described above.

For hKir3.1/3.4, antazoline exhibited a concentration-dependent inhibition with a calculated half-maximal inhibitory concentration (IC_50_) of 52.8 µM and a Hill coefficient (*n*_*H*_) of −1.3 (*n =* 5; Figure 2A). In the case of hERG, distinct IC_50_ values were calculated for the two current components. The end-pulse current showed a more potent inhibition, with an IC_50_ of 43.3 µM and a Hill coefficient of −0.9 (*n =* 4–6; Figure 2B). In contrast, the peak tail current component was less sensitive, displaying an IC_50_ of 162.6 µM with a Hill coefficient of −1.4 (*n =* 4–6; Figure 2C).

These results indicate that antazoline inhibits both hK_ir_3.1/3.4 and hERG currents in a concentration-dependent manner, with a higher potency toward hK_ir_3.1/3.4 and the end-pulse component of hERG.

### 3.3 Inhibition of hK_ir_3.1/3.4 channel heteromers by antazoline

**FIGURE 3:**
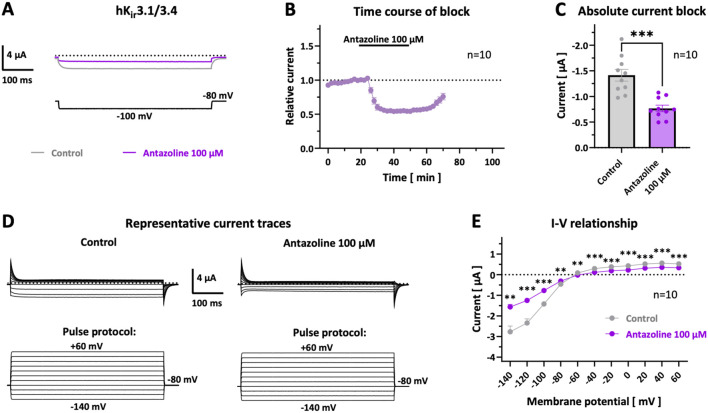
*Modulation of hK*_*ir*_ *3.1/3.4 currents by antazoline.* **(A)** Representative current traces recorded at −100 mV under control conditions (grey) and following 30 min incubation with 100 µM antazoline (purple). Dotted lines represent the zero current level and scalebars are provided as insets. **(B)** Time course of relative current inhibition at −100 mV during antazoline perfusion and washout (*n =* 10 cells). **(C)** Bar graph showing absolute current amplitudes at −100 mV before (grey) and after (purple) 30 min antazoline application (*n =* 10 cells). **(D)** Representative current traces recorded at test potentials from −140 to +60 mV in 20 mV increments under control conditions and following 30 min antazoline perfusion. **(E)** Current–voltage (I–V) relationship of hK_ir_3.1/3.4-mediated currents under control conditions (grey) and after 30 min antazoline exposure (purple), corresponding to the voltage steps shown in **(D)** (*n =* 10 cells). Data are provided as mean ± SEM of n = 10 measurements; *, *p <* 0.05; **, *p <* 0.01; ***; *p <* 0.001 derived from single **(C)** or multiple **(E)** two-tailed Student’s t-tests, followed by Bonferroni-Dunn correction.

To gain a more detailed understanding of the inhibitory effects of antazoline on hK_ir_3.1/3.4 channels (Figure 3A), the time course of current suppression was monitored (Figure 3B). After a control stabilization period of at least 20 min in external bath solution, perfusion with bath solution containing 100 µM antazoline was initiated. This resulted in a rapid decline in current amplitude after the first 10–14 min of superfusion. At a holding potential of −80 mV, 30-min antazoline treatment significantly reduced mean current amplitudes from −1.4 ± 0.1 µA to −0.8 ± 0.1 µA by 45.8% (*p <* 0.0001; *n =* 10; Figures 3A,C).

As shown in Figure 3B, the antazoline-induced current block was partially reversible. Following a 20 min washout period in drug-free solution, current amplitudes recovered to 75.8% ± 4.8% of baseline levels (*n =* 10), indicating substantial but incomplete reversibility of hK_ir_3.1/3.4 channel inhibition by antazoline. Figures 3D,E illustrate the antazoline-induced current suppression across all tested membrane potentials, leading to a pronounced flattening of the current–voltage (I–V) relationship. The inhibition was statistically significant at all test potentials.

### 3.4 Inhibition of hERG currents by antazoline

**FIGURE 4:**
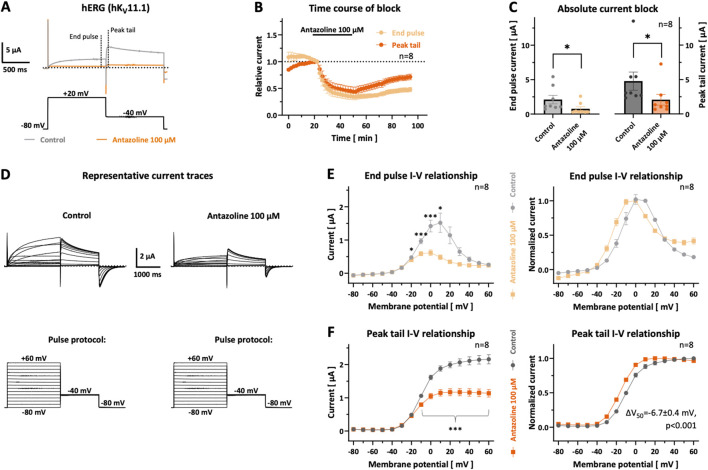
*hERG current inhibition by antazoline.* **(A)** Representative current traces recorded under control conditions (grey) and after 30-min incubation with 100 µM antazoline (orange), shown with the corresponding double-step pulse protocol. **(B)** Time course of antazoline-induced relative current inhibition (100 μM; 30 min as indicated) as well as washout (44 min), measured for both the end-pulse (i.e., the steady-state/depolarization current; light orange) and the peak-tail (repolarizing current; dark orange) current components (*n =* 8 cells). **(C)** Scatter plot of absolute end-pulse and peak-tail current amplitudes before (light and dark grey) and after (light and dark orange) 30 min antazoline treatment (*n =* 8 cells). **(D)** Representative macroscopic current traces illustrating hERG voltage-dependent activation under control conditions and following 30 min antazoline perfusion, together with the applied pulse protocol. **(E)** End-pulse current–voltage (I–V) relationships under control conditions (light grey) and after antazoline exposure (light orange); left panel: absolute current amplitudes; right panel: values normalized to the respective maximal current (*n =* 8 cells). **(F)** Peak-tail I–V relationships under control conditions (dark grey) and after antazoline incubation (dark orange); left panel: absolute amplitudes; right panel: normalized values (*n =* 8 cells). The mean half-maximal activation voltage (*V*_50_) was significantly shifted by −6.7 ± 0.4 mV toward more negative potentials (peak-tail currents were normalized to the maximal current amplitude per cell prior to Boltzmann fitting on a per-cell basis). Dotted lines indicate the zero current level, pulse protocols are depicted below the respective current traces and scalebars are provided as insets. Data are presented as mean ± SEM of *n =* 8 cells. Statistical significance, determined from multiple paired two-tailed t-tests, if necessary, followed by Bonferroni-Dunn correction, is indicated as *, *p <* 0.05; **, *p <* 0.01; ***, *p <* 0.001.

In analogy to hK_ir_3.1/3.4, the modulatory effects of antazoline on hERG channels were further investigated. Drug effects were analyzed for two distinct current components: the end-pulse current, reflecting depolarized steady-state conditions, and the peak-tail current, representing repolarization-induced recovery from inactivation (Figure 4A). The time course of current inhibition by 100 µM antazoline is shown in Figure 4B, using the same experimental conditions and the double-step voltage protocol described above. Antazoline induced a rapid inhibition of both hERG current components. As shown in Figure 4C, end-pulse current amplitudes were significantly reduced from 2.1 ± 0.6 µA to 0.8 ± 0.3 µA (*p =* 0.0252; *n =* 8), and peak-tail currents from 4.8 ± 1.3 µA to 2.1 ± 0.8 µA (*p =* 0.0171; *n =* 8) after 30 min of antazoline application. While Figure 4B suggests a slightly stronger reduction in the end-pulse component, no statistically significant difference between end-pulse and peak-tail inhibition was detected (end-pulse block: 65.8% ± 5.5%; peak-tail block: 56.1% ± 8.6%; *p =* 0.0650; *n =* 8).

Following drug washout, partial recovery of hERG currents was observed (Figure 4B). End-pulse current levels recovered to 38.4% ± 3.4% (*p =* 0.3025; *n =* 8) of baseline after 20 min and to 47.9% ± 4.1% (*p =* 0.0242; *n =* 7) after 44 min. For the peak-tail component, recovery was even more pronounced, reaching 58.2% ± 6.2% (*p =* 0.1071; *n =* 8) at 20 min and 71.5% ± 5.2% (*p =* 0.0277; *n =* 7) at 44 min. However, the difference in recovery between the two components did not reach statistical significance at either time point (20 min: *p =* 0.1833, *n =* 8; 44 min: *p =* 0.0994, *n =* 7).

### 3.5 Antazoline effects on the voltage-dependence of hERG and its activation

The effects of 100 µM antazoline on hERG channel activation were evaluated by analyzing drug-induced changes in the I–V relationship (Figures 4D–F). hERG channels were activated by 2.2 s depolarizing pulses from −80 to +60 mV in 10 mV increments (0.1613 Hz), followed by repolarization to −40 mV for 1.6 s. I–V curves were constructed from recordings obtained after ≥20 min of stabilization under control conditions and following 30 min antazoline superfusion. For this purpose, end-pulse (Figure 4E) and peak-tail (Figure 4F) current components were quantified as indicated in Figure 4A and plotted as a function of the respective test pulse potential and no leak current subtraction was applied. For the depiction of normalized current–voltage relationships (Figures 4E,F, right-hand side), absolute current amplitudes were normalized to the respective maximal current recorded in each individual cell.

Under control conditions, end-pulse currents began to activate at voltages ≥ −50 mV, peaked between 0 and +10 mV, and declined at more positive potentials. In contrast, following antazoline treatment, the maximum of the end-pulse I–V curve shifted to more negative voltages, between −10 and 0 mV. Significant current reduction by antazoline was observed within the range of −20 to +10 mV (Figure 4E). The voltage dependence of peak-tail currents followed a sigmoidal activation pattern under both conditions (Figure 4F). Antazoline significantly reduced peak-tail amplitudes at voltages more positive than −20 mV. At +60 mV, the degree of inhibition reached 48.1% ± 2.2% (*p <* 0.0001; *n =* 8). Moreover, antazoline significantly shifted the half-maximal activation potential (*V*_50_) from −9.4 ± 0.9 mV to −16.1 ± 0.9 mV (*p <* 0.0001; *n =* 8), shifting channel activation at more negative voltages.

### 3.6 State dependence of hERG inhibition by antazoline

**FIGURE 5:**
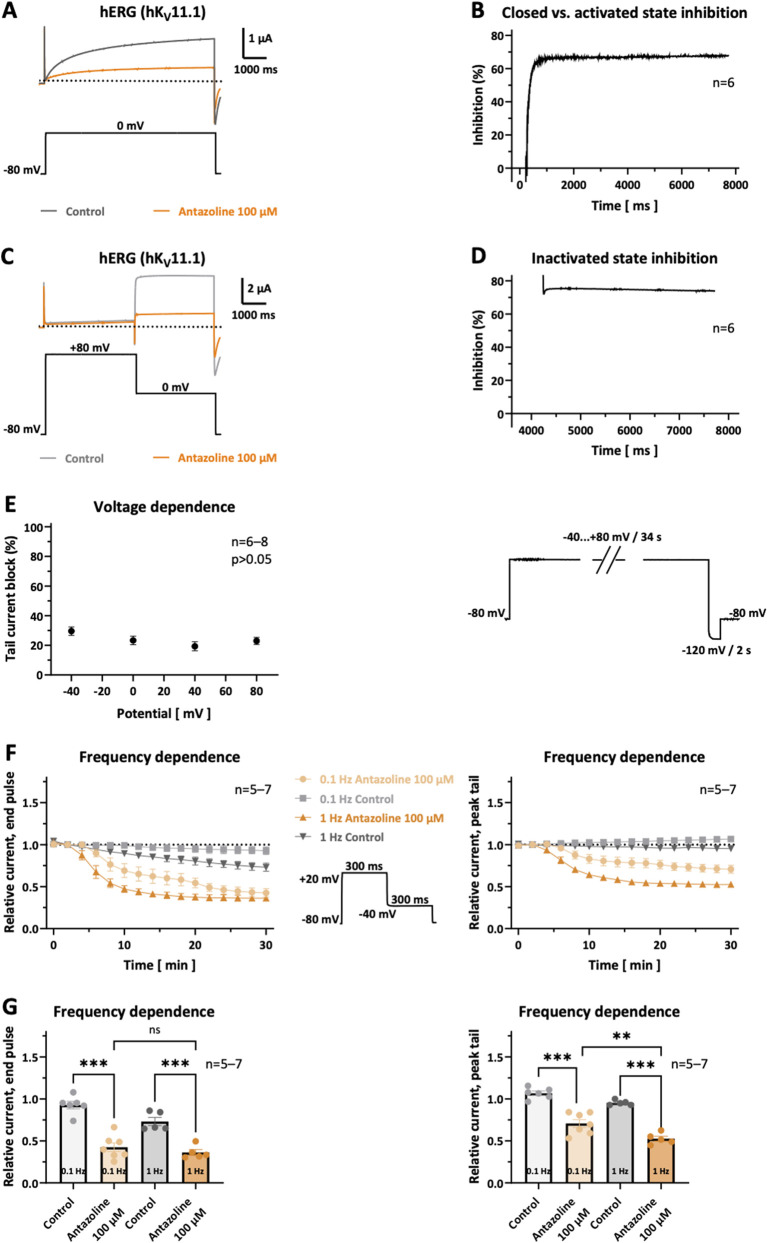
*Biophysical characterization of hERG channel blockade by antazoline.* **(A,B)** Block of hERG channels in the activated state by antazoline. **(A)** Representative current traces were recorded under control conditions and after 30 min of superfusion with 100 µM antazoline, during which the oocyte was clamped at −80 mV. The control recording (grey) and the first pulse measured after the incubation period (orange) are displayed. **(B)** Time course of current inhibition under the same conditions. Current inhibition increased time-dependently, illustrating that channel block occurs in the activated (i.e., the open and/or the inactivated channel state) and not in the preceding closed state. Similar results were observed in *n =* 6 experiments. **(C,D)** Assessment of hERG inhibition by antazoline in the inactivated state (*n =* 6 cells). **(C)** hERG channels were first driven into the inactivated state by a depolarizing step to +80 mV, followed by a second step to 0 mV to reopen the channels. The corresponding relative current inhibition during the 0 mV step is shown in **(D)**. Representative current traces before (grey) and after (orange) 30 min antazoline application, along with the corresponding voltage protocol. A rapid onset of block (∼74%) was observed, indicating that antazoline binds preferentially to the inactivated state of the channel, with only a minimal additional increase in inhibition upon channel reopening. **(E)** Antazoline-induced block of hERG channels is voltage-independent. *Left*: Peak-tail current inhibition plotted against the test potential after 30 min antazoline perfusion (*n =* six to eight cells per condition). *Right*: Voltage protocol used to assess voltage-dependent block. No significant difference in the degree of block was observed across membrane potentials from −40–80 mV. **(F)** Frequency dependence of hERG inhibition by antazoline, stratified for end-pulse current reduction (*left*) and peak-tail current reduction (*right*). Currents were recorded in the presence of antazoline at an activation rate of 0.1 Hz (light orange) and 1 Hz (dark orange), with corresponding control traces at 0.1 Hz (light grey) and 1 Hz (dark grey; *n =* five to seven individual cells per condition). In both current components, the onset of antazoline-induced current block was more rapid at 1 Hz compared to 0.1 Hz. **(G)** Frequency dependence of antazoline-induced hERG current inhibition (100 μM; 30 min; *n =* five to seven cells per condition). At a stimulation rate of 1 Hz antazoline application led to significantly stronger inhibition of peak-tail currents (*right*) compared to 0.1 Hz, while no significant difference was detected for end-pulse currents (*left).* Dotted lines indicate the zero current level. Corresponding pulse protocols are depicted below the respective current traces. Scalebars are provided as insets. Data are presented as mean ± SEM. Statistical significance, derived from one-way ANOVA with Tukey’s multiple comparisons test: ns, not significant; *, *p* < 0.05; **, *p <* 0.01; ***, *p <* 0.001.

To investigate the state-dependent characteristics of hERG channel blockade by antazoline, modified voltage-clamp protocols were applied to isolate drug effects in the closed, open, and inactivated channel states. In both experimental conditions, baseline control measurements were obtained every 2 minutes during a stabilization period of ≥20 min. Antazoline (100 µM) was then applied while the channels were held in the closed state at −80 mV for 30 min. At the end of the incubation period, the respective pulse protocol was re-applied, and the degree of current inhibition was calculated using the following formula: Inhibition (%) = (1 – *I*_antazoline_/*I*_control_) × 100, where *I* represents the measured current amplitude. To distinguish between closed-state and activated-state (i.e., open and/or inactivated) block, the first protocol involved a single 7.5 s depolarizing pulse to 0 mV, followed by a return to the −80 mV holding potential (Figure 5A). As shown in Figure 5B, antazoline induced a time-dependent current reduction upon channel activation illustrating that channel block occurs during activated and not in the preceding closed state. This finding is consistent with inhibition of hERG potassium channels in the activated state, albeit a contribution of closed-state block cannot be fully excluded based on this protocol. In this experimental series, outward hERG currents measured at the end of the 0 mV pulse were reduced by 63.1% ± 3.0% (*n* = 6; Figure 5B).

To further differentiate the activated state between open-state and inactivated-state block, a second protocol was employed (Figure 5C). This consisted of a 4 s depolarizing step to +80 mV to inactivate the channels, immediately followed by a 3.5 s test pulse to 0 mV to reopen the channels. As shown in Figure 5D, only a minimal time-dependent increase in inhibition was observed during the opening phase. The mean inhibition at the end of the 0 mV step was 74.7% ± 2.0% (*n =* 6), suggesting that most of the block had already occurred during inactivation.

### 3.7 Voltage dependence of hERG block by antazoline

To assess the voltage dependence of hERG channel inhibition, currents were recorded before and after 30 min perfusion with 100 µM antazoline, during which channels were held in the closed state at −80 mV. A voltage-clamp protocol consisting of a prolonged 34 s depolarizing step to either −40, 0, +40, or +80 mV was applied, followed by a 2 s repolarization to −120 mV to elicit inward peak-tail currents. Each test pulse voltage was applied to a separate set of oocytes. The extent of antazoline-induced inhibition was calculated from the ratio of peak-tail current amplitudes recorded under control and drug conditions. As shown in Figure 5E, no statistically significant difference in the degree of current inhibition was observed across the voltage range tested (*n =* six to eight cells per voltage), indicating a voltage-independent blocking profile under these conditions.

### 3.8 Frequency dependence of hERG block by antazoline

The frequency dependence of hERG inhibition by antazoline was evaluated by comparing current responses under low-frequency (0.1 Hz) and high-frequency (1 Hz) stimulation protocols. Each protocol consisted of a 300 ms depolarization to +20 mV followed by a 300 ms repolarization to −40 mV. Stimulation and rest phases alternated every minute, and in each active stimulation phase, the final sweep (60th for 1 Hz, sixth for 0.1 Hz) was analyzed. Cells were exposed to either 100 µM antazoline or drug-free bath solution, following a baseline stabilization period. As depicted in Figures 5F,G, both hERG current components, end-pulse as well as peak-tail, exhibited a more rapid onset of antazoline-induced current inhibition under 1 Hz stimulation as compared to 0.1 Hz.

At the end of the 30 min recording period, the relative block of end-pulse currents did not differ significantly between frequencies (0.1 Hz: 57.5% ± 5.2%, *n =* 7; 1 Hz: 63.8% ± 3.5%, *n =* 5; *p =* 0.7845). In contrast, the peak-tail current block was significantly stronger in the 1 Hz group compared to the 0.1 Hz group (0.1 Hz: 29.4% ± 4.7%, *n =* 7; 1 Hz: 47.4% ± 3.0%, *n =* 5; *p =* 0.0088), confirming a frequency-dependent component in antazoline’s hERG inhibition for peak-tail currents.

### 3.9 Molecular determinants of antazoline–hERG interaction

**FIGURE 6:**
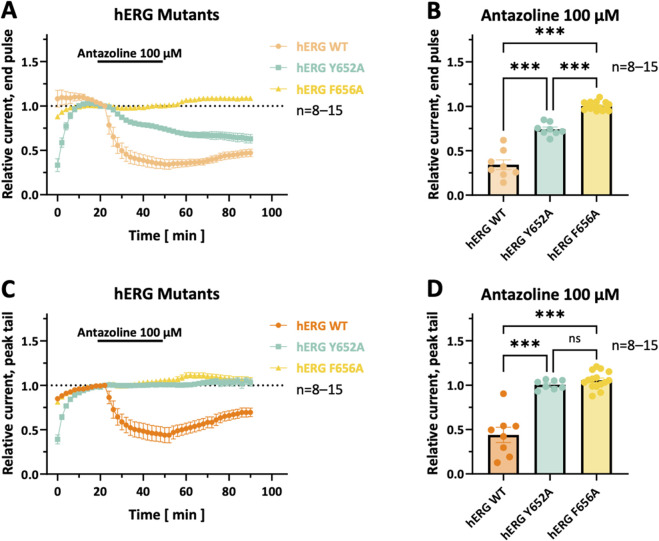
*Effects of Y652A and F656A mutations on hERG channel inhibition by 100 µM antazoline.* **(A)** Time course of end-pulse current inhibition by 100 µM antazoline in hERG wild-type (WT) (light orange), hERG-Y652A (blue), and hERG-F656A (yellow) channels. **(B)** Relative end-pulse current amplitudes after 30 min of antazoline perfusion are depicted for hERG-WT, hERG-Y652A, and hERG-F656A channels (*n =* 8–15 cells, respectively). **(C)** Time course of peak-tail current inhibition by 100 µM antazoline in hERG-WT (dark orange), hERG-Y652A (blue), and hERG-F656A (yellow) channels. **(D)** Respective relative peak-tail current amplitudes after 30 min antazoline (100 µM) application. Both hERG-Y652A and hERG-F656A mutations significantly attenuated the inhibitory effect of antazoline on hERG currents. Dotted lines indicate the zero current level. Values are presented as mean ± SEM. Statistical significance, derived from one-way ANOVA with Tukey’s multiple comparisons test: ns = not significant; *, *p <* 0.05; **, *p <* 0.01; ***, *p <* 0.001.

The S6 pore-forming domain of the hERG channel harbors two aromatic residues, tyrosine 652 (Y652) and phenylalanine 656 (F656), which are considered critical for molecular drug binding interactions (Mitcheson et al., 2000). To assess their role in antazoline binding, the inhibitory effects of the drug were compared among hERG-WT channels and on channels carrying point mutations at these positions (Y652A and F656A). Cells were superfused with 100 µM antazoline for 30 min, and hERG currents were elicited using the same double-step voltage-clamp protocol as described above (Figure 6).

For end-pulse currents, antazoline induced a relative inhibition of 65.8% ± 5.5% in hERG-WT channels (*n =* 8). In contrast, this effect was reduced to 25.8% ± 2.5% in hERG-Y652A mutants (*p <* 0.0001; *n =* 8; Figures 6A,B), and almost completely abolished in hERG-F656A mutants, which exhibited a negligible block of −0.3% ± 1.2% (*p <* 0.0001; *n =* 15; Figures 6A,B). A similar pattern was observed for the peak-tail currents. Here, hERG-WT channels showed a relative block of 56.1% ± 8.6% (*n =* 8), while the hERG-Y652A and hERG-F656A mutants showed strongly reduced inhibition, with values of −0.6% ± 1.8% (*p <* 0.0001; *n =* 8; Figures 6C,D) and −5.6% ± 2.5% (*p <* 0.0001; *n =* 15; Figures 6C,D), respectively.

These findings indicate that both Y652 and F656 are crucial for antazoline-mediated hERG channel blockade, with F656 playing an even more dominant role.

## 4 Discussion

Antazoline, a first-generation H_1_-antihistamine, has long been recognized for its antiarrhythmic effects, particularly the acute pharmacological cardioversion of pAF. Recent clinical trials have demonstrated conversion rates ranging from 63% to 85%, comparable to those of propafenone and significantly exceeding those of amiodarone, with additional advantages of rapid onset and favorable tolerability (Maciag et al., 2017; Karwowski et al., 2024; Wybraniec et al., 2018; Wybraniec et al., 2022). These observations have renewed interest in antazoline as a candidate for antiarrhythmic drug repurposing. However, the underlying electrophysiological mechanisms remain incompletely understood.

### 4.1 Pharmacological profile and key targets of antazoline

hK_ir_3.1/3.4 channels form the molecular correlate of *I*_K,ACh_ and possess a cytoplasmic pore architecture that enables interaction with cationic amphiphilic compounds. Although specific binding residues were not dissected in the present study, the rapid and partially reversible inhibition observed is consistent with pore-associated or cytoplasmic vestibule interactions described for other antiarrhythmic agents acting on GIRK channels. Given the predominantly atrial expression pattern and pathophysiological relevance of hK_ir_3.1/3.4 in atrial fibrillation, its inhibition represents a plausible contributor to the atrial electrophysiological effects of antazoline (Seebohm, 2009).

In contrast, hERG was selected for detailed biophysical and mutational analysis due to its central role in ventricular repolarization and its well-established involvement in drug-induced long QT syndrome. Moreover, hERG/*I*_Kr_ constitutes a key molecular target of several Class III antiarrhythmic drugs used for rhythm control in atrial fibrillation, including dofetilide, sotalol, and amiodarone. Even moderate hERG inhibition may therefore have both therapeutic and safety implications. For this reason, structural determinants, state dependence, and frequency dependence of hERG block were investigated in greater mechanistic depth.

Other channels, including hK_2P_9.1, hK_2P_18.1, hK_V_1.5 and hK_V_LQT1/MinK showed modest inhibition, while hK_2P_17.1 and rK_V_1.4 were significantly activated. As hK_2P_17.1 is known to be expressed in the human heart, predominantly in atrial and Purkinje fibers (Wiedmann et al., 2021), it might contribute to antazoline’s cardiac electrophysiological profile.

### 4.2 Biophysical characteristics of hERG channel inhibition

Detailed biophysical analyses demonstrated that antazoline inhibits hERG currents in a state- and frequency-dependent manner. Time-dependent current suppression was observed during depolarizing pulses, indicating preferential binding to the open or inactivated states of the channel. This interpretation was further supported by protocols specifically designed to separate open-state from inactivated-state contributions. During sustained depolarization, a rapid onset of current block was detected, consistent with inhibition occurring after channel activation. Subsequent reopening from the inactivated state produced only minimal additional inhibition, suggesting that antazoline binding had largely occurred during inactivation. Furthermore, the frequency dependence of block, particularly for peak-tail currents, supports a model of use-dependent inhibition facilitated by channel state transitions during repetitive stimulation which is also consistent with blockade of open and inactivated channels.

### 4.3 Structural determinants of hERG block by antazoline

The S6 transmembrane segment of the hERG channel forms a central part of the drug-binding cavity and contains two highly conserved aromatic residues, tyrosine 652 (Y652) and phenylalanine 656 (F656), which are recognized as critical determinants of high-affinity binding. In the present study, alanine substitution of either residue markedly attenuated antazoline-induced current inhibition. While mutation of Y652 reduced end-pulse and peak-tail block to 25.8% and −0.6%, respectively, substitution of F656 almost completely abolished antazoline-mediated inhibition. These findings demonstrate that both residues are critical for antazoline binding, with F656 playing a dominant role.

The chemical structure of antazoline harbors planar aromatic rings and protonatable nitrogens, particularly in the imidazoline ring. It is therefore plausible that antazoline engages in π-stacking with F656 and cation–π bonding with Y652, stabilizing its position within the central cavity of the channel in a conformation-dependent manner. The requirement for these specific residues further supports a direct pore-binding mechanism, consistent with the observed state- and frequency-dependent electrophysiological properties. Notably, structurally related H1-antihistamines such as terfenadine and astemizole, both withdrawn from the market due to QT prolongation and torsades de pointes (TdP), also interact with Y652 and F656 (Mitcheson et al., 2000; Miyashita et al., 2024). However, compared to these agents, antazoline has not been associated with a comparable incidence of ventricular arrhythmias in clinical studies.

One possible explanation for the different clinical safety profiles may lie in the markedly higher affinity of these compounds for the hERG channel. Terfenadine and astemizole have been reported to inhibit hERG with IC_50_ values in the high-to low-nanomolar range, indicating high-affinity block (Suessbrich et al., 1996; Kamiya et al., 2008), whereas antazoline, based on the present data, constitutes a moderate-to low-affinity inhibitor in the micromolar range. Although direct cross-system comparisons must be interpreted cautiously, the lower apparent affinity of antazoline for hERG may contribute to its comparatively more favorable ventricular safety profile observed in clinical settings.

### 4.4 Electrophysiological implications of multichannel inhibition

The electrophysiological effects of antazoline observed in this study support a multi-channel mechanism that may account for its clinically effective suppression of atrial arrhythmias. Inhibition of hK_ir_3.1/3.4 channels, which underlie the acetylcholine-regulated inward rectifier potassium current (*I*_K,ACh_), is particularly relevant, as these channels are predominantly expressed in the atria and contribute to action potential (AP) shortening and reentry stabilization under vagal stimulation (Yamada et al., 1998). In cardiomyocytes from permanent AF patients, the hK_ir_3.1/3.4 channels are reported to be downregulated on expression level, yet developing constitutive activity (Dobrev et al., 2001; Dobrev et al., 2005). Many antiarrhythmic drugs are shown to suppress hK_ir_3.1/3.4 currents, including amiodarone (Watanabe et al., 1996), dofetilide (Voigt et al., 2010), dronedarone (Altomare et al., 2000), propafenone (Voigt et al., 2010) and sotalol (Mori et al., 1995). By suppressing *I*_K,ACh_, antazoline may prolong the atrial APD and increase the ERP, thereby reducing the substrate for AF maintenance. Furthermore, hK_ir_3.1/3.4 blockade was also described for the antihistamine terfenadine (Chen et al., 2019).

In parallel, potent inhibition of hERG currents by antazoline implicates the delayed rectifier potassium current (*I*_Kr_), which governs phase 3 repolarization of the cardiac AP in both atria and ventricles. Suppression of *I*_Kr_ prolongs APD and ERP and contributes to the antiarrhythmic efficacy of Class III agents. However, excessive *I*_Kr_ inhibition is also associated with delayed repolarization, QT interval prolongation, and increased risk of early afterdepolarizations (EADs) and TdP. While hERG block was clearly evident in this study, the positive rate dependence of antazoline inhibition, more pronounced at 1 Hz than at 0.1 Hz, suggests use-dependent behavior, generally considered favorable in treatment of tachyarrhythmias (Kiesecker et al., 2004; Kiehn et al., 1999). However, this property alone does not preclude proarrhythmic effects, as similar behavior has also been observed with the withdrawn antihistamines terfenadine and astemizole (Suessbrich et al., 1996). Thus, antazoline-mediated hERG inhibition may confer both antiarrhythmic benefits and proarrhythmic risks.

In addition to *I*_Kr_ inhibition, antazoline (100 µM) produced approximately 30% suppression of hK_V_LQT1/MinK currents (*I*_Ks_). Although this effect was less pronounced than hERG block, partial *I*_Ks_ inhibition may contribute to atrial APD prolongation. While *I*_Ks_ plays a major role in ventricular repolarization reserve, it is also expressed in atrial myocardium, where it participates in late phase 3 repolarization, particularly under β-adrenergic stimulation. Moderate *I*_Ks_ inhibition may therefore enhance atrial ERP prolongation in situations of increased sympathetic tone, a common trigger of paroxysmal AF. In the setting of concomitant *I*_K,ACh_ and *I*_Kr_ inhibition, additional *I*_Ks_ suppression may reinforce atrial repolarization delay and stabilize sinus rhythm.

Taken together, the combined inhibition of atrial-selective currents (*I*_K,ACh_), universal repolarizing currents (*I*_Kr_ *, I*_Ks_), and concomitant background current activation suggests a multifaceted modulation of cardiac electrophysiology by antazoline. This profile aligns with the emerging concept of multichannel blockade as a desirable strategy for rhythm control, particularly when atrial-selectivity and balanced repolarization are prioritized to limit ventricular proarrhythmia.

In former studies, antazoline-mediated inhibition of hERG has been demonstrated in HEK293 cells using the whole-cell patch clamp technique, yielding an IC_50_ of approximately 3 µM (Badyra et al., 2017). Indirect evidence of hERG blockade has also emerged from an experimental rabbit heart model of acquired long QT syndrome (LQTS) (Ellermann et al., 2018), where antazoline induced more pronounced QT prolongation in LQTS3 hearts than in LQTS2 hearts (pretreated with *I*_Kr_ blockers). In agreement with our results, the study further documented positive rate dependence of antazoline action. Additionally, antazoline has been reported to inhibit *I*_K,ATP_ channels in single-channel inside-out patch recordings from guinea pig ventricular cardiomyocytes, with 10 µM causing 68.4% ± 7.3% inhibition (Lee et al., 1995). Although *I*_K,ATP_ channel screening was not included in our study, determination of an IC_50_ value would be essential to assess whether antazoline significantly interferes with the cardioprotective, membrane-stabilizing role of *I*_K,ATP_ channels during ischemic conditions.

### 4.5 Clinical implications and safety considerations

The inhibition of hK_ir_3.1/3.4 and hERG observed here is consistent with clinical findings of P-wave, QRS, and QTc interval prolongation following intravenous administration, indicating effects on both atrial conduction and ventricular repolarization (Maciag et al., 2017; Karwowski et al., 2024; Piotrowski et al., 2017; Wybraniec et al., 2018; Wybraniec et al., 2022).

A key consideration in translating *in vitro* findings to clinical practice is the interpretation of IC_50_ values obtained in *X. laevis* oocytes. While the oocyte system provides robust assessment of state dependence, mutation sensitivity, and qualitative channel pharmacology, absolute potency estimates may deviate substantially from mammalian systems. Due to diffusion barriers imposed by the vitelline membrane and yolk content, apparent IC_50_ values in *Xenopus oocytes* have been reported to be 5–30-fold higher compared with mammalian expression systems, depending on compound properties and channel type (Madeja et al., 1997; Kiesecker et al., 2004; Gierten et al., 2008; Wiedmann et al., 2022). Accordingly, the IC_50_ values determined in the present study should be interpreted as system-dependent estimates rather than definitive potency values for the human myocardium.

In human healthy volunteers, plasma peak antazoline concentrations of approximately 11.3 µM were measured after a cumulative intravenous dose of 300 mg administered in 100 mg boluses (Piotrowski et al., 2017), with plasma protein binding reported to be <50% (Wiśniowska et al., 2022). Moreover, metabolization of antazoline via CYP2D6 was described (Giebułtowicz et al., 2020), allowing for a wide interindividual range of plasma levels due to common genetic polymorphisms and drug interactions (Ingelman-Sundberg, 2005). Notably, previously reported IC_50_ values for hERG inhibition in HEK293 cells around 3 µM (Badyra et al., 2017) are within the range of measured clinical plasma concentrations. This observation supports the notion that hERG modulation by antazoline may occur at therapeutically relevant exposure levels and therefore carries potential clinical implications. The higher IC_50_ values obtained in the present *Xenopus system* are consistent with known diffusion-related overestimations in oocytes as discussed above.

Nevertheless, direct quantitative extrapolation from *in vitro* systems to human myocardial pharmacodynamics remains speculative. Therefore, conclusions regarding therapeutic safety margins or proarrhythmic thresholds should be regarded as hypothesis-generating rather than definitive.

Repeated reports of QT interval prolongation in humans following antazoline administration have raised safety considerations (Piotrowski et al., 2017; Bińkowski et al., 2018). However, to our knowledge, no cases of TdP have been documented in the literature following intravenous antazoline infusion (Karwowski et al., 2024; Maciag et al., 2017; Piotrowski et al., 2017; Wybraniec et al., 2018; Wybraniec et al., 2022; Farkowski et al., 2018; Calvert et al., 2022; Palimonka et al., 2020). This contrasts with structurally related antihistamines like terfenadine (Mitcheson et al., 2000) and astemizole (Miyashita et al., 2024), which were withdrawn due to significant QT prolongation and TdP risk. As discussed above, these agents exhibit substantially higher affinity for hERG channels compared with antazoline, which may partly account for the differing clinical safety profiles. Nevertheless, careful evaluation of ventricular repolarization effects remains warranted.

### 4.6 Potential limitations

The findings of this study should be interpreted within the limitations of the *X. laevis* oocyte expression system. This heterologous model enables high signal-to-noise recordings and precise biophysical dissection of state dependence, frequency dependence, and mutation-sensitive binding mechanisms. However, absolute IC_50_ values obtained in oocytes may differ markedly from those measured in mammalian cells or native cardiomyocytes. As described above, the vitelline membrane and intracellular yolk content can limit compound diffusion and lead to overestimation of apparent inhibitory concentrations. Reported differences between *Xenopus* and mammalian systems range from 5-fold up to 30-fold, depending on compound lipophilicity and membrane permeability (Madeja et al., 1997; Kiesecker et al., 2004; Gierten et al., 2008; Wiedmann et al., 2022). Consequently, concentration–effect relationships described here should not be interpreted as direct predictors of human myocardial pharmacodynamics.

Furthermore, the concept of atrial selectivity derived from multichannel inhibition remains inferential. Although hK_ir_3.1/3.4 blockade and hK_2P_17.1 activation may preferentially affect atrial electrophysiology, the present experiments were not performed in human atrial cardiomyocytes and therefore cannot establish functional atrial specificity. Future studies in mammalian systems, native human tissue and complementary *in silico* modelling approaches, such as human action potential simulations in accordance with the Comprehensive *in vitro* Proarrhythmia Assay (CiPA) initiative, would be valuable to further define the integrated electrophysiological effects and proarrhythmic risk profile of antazoline.

### 4.7 Conclusion

This study identifies hK_ir_3.1/3.4 and hERG as principal electrophysiological targets of antazoline and demonstrates a concentration-dependent, partially reversible inhibition of both channels. Additional activation of atrial-selective hK_2P_17.1 suggests a multi-channel mode of action that may contribute to antazoline’s antiarrhythmic effects on atrial myocardium. While these findings provide mechanistic support for the repurposing of antazoline as a rhythm control agent, quantitative translation of potency and safety margins from the *Xenopus* system to human myocardium remains speculative. Further validation in mammalian models and human cardiomyocytes is required to define its atrial–ventricular selectivity and clinical safety profile more precisely.

## Data Availability

The raw data supporting the conclusions of this article will be made available by the authors, without undue reservation.
